# Physical Characteristics of Sintered Silver Nanoparticle Inks with Different Sizes during Furnace Sintering

**DOI:** 10.3390/ma17050978

**Published:** 2024-02-20

**Authors:** Hyeong-Jin Park, Kyongtae Ryu, Hee-Lak Lee, Yoon-Jae Moon, Jun Young Hwang, Seung Jae Moon

**Affiliations:** 1 Department of Mechanical Convergence Engineering, Hanyang University, Seoul 04763, Republic of Korea; gudwlsrns87@gmail.com (H.-J.P.); ktrzzang@hanyang.ac.kr (K.R.); joylee1112@hanyang.ac.kr (H.-L.L.); myj3235@kitech.re.kr (Y.-J.M.); 2 Korea Institute of Industrial Technology, Ansan 15558, Republic of Korea; jyhwang@kitech.re.kr

**Keywords:** silver nanoparticle ink, furnace sintering, specific resistance, surface morphology

## Abstract

The influence of nanoparticle (NP) size on the physical characteristics of sintered silver NP ink was studied using four different types of inks. The Ag NP inks were spin-coated on glass substrates with an average thickness of 300 nm. Each sample was sintered for 30 min, with temperatures from 50 °C to 400 °C by an interval of 50 °C. After sintering, the specific resistance of each case was obtained using the resistance and surface profile measurements. The minimum specific resistance obtained by the experiment was 2.6 μΩ·cm in the case in which 50 nm-sized Ag NP ink was sintered at 350 °C. The transformed surface morphology and grain size of each case were observed using scanning electron microscopy and atomic force microscopy. The results of this study can be a reference for future manufacturers in selecting the Ag NP size and the sintering temperature.

## 1. Introduction

The conventional manufacturing process of electrical and electronic devices is mainly composed of doping, deposition, photolithography, and etching processes. The doping process gives electrical functions to intrinsic wafers by injecting impurity atoms and the deposition process adds a layer of functional material on the wafer surface. The photolithography process creates a pattern on the wafer surface using a mask and the etching process uses acid solutions in wet etching or plasma in dry etching to remove unnecessary parts of the wafer patterned with photoresist. However, these processes require high-temperature or high-vacuum-processing environments and have a high cost due to complex processing procedures. Moreover, material loss due to etching is significant, and the toxic waste due to the use of highly acidic solutions in wet etching processes causes high environmental loads [1,2]. In contrast to the conventional manufacturing process, the printed electronics manufacturing process has only three stages, i.e., printing, sintering, and inspection. The simple manufacturing process of printed electronics reduces the time for designing and manufacturing, as well as the unit cost. Moreover, it does not produce toxic waste because of the fact that photolithography and etching processes are not required [3].

In printed electronics, the electric circuit of an electric device is manufactured by printing conductive ink on the substrate. When using metal nanoparticle (NP) inks, the van der Waals force between the metal NPs can cause agglomeration, resulting in poor print quality [4]. A dispersant is included in the metal NP inks to prevent such a phenomenon. The presence of a dispersant disrupts the movement of electrons, and it causes the resistance of the printed circuits to increase. A thermal sintering process is needed to reduce the specific resistance by removing the dispersant and to improve the connectivity between metal NPs [5]. The sintering temperature is known to be an important factor. The increase in the sintering temperature enhances neck formation in the particle boundary, which boosts the connection between particles and leads to densification. This is because the reduction of surface energy influences the neck formation in the particle boundary [6].

The sintering process of metal nanoparticle inks have been studied extensively in recent years [7,8,9]. Many studies have been conducted with different sintering methods such as furnace [10], hotplate [11,12,13], laser [14], electrical [15], plasma [16,17], microwave sintering [18,19], etc. Studies have investigated the effect of sintering conditions, i.e., sintering temperature [20], heating time [21,22], parameter configurations [22,23,24], etc., on the sintering results. The size of the NP has also been studied as an influential factor on the characteristics of the sintered NPs [25]. The NPs with different sizes have different sintering characteristics because they have different surface energies [26]. Studies have suggested that the surface energy leads to differences in melting temperature [27,28,29,30,31,32], optical properties [9], and the suggested sintering method described by [21]. Bochnia [33] investigated the effects of the natural aging period on the tensile strength and elastic modulus in plastics. Baroutaji et al. [34] examined the effects of atmospheric oxygen content on the mechanical properties of selectively laser-melted AlSi_10_Mg. Formisano et al. [35] optimized the single-point incremental forming process for polymer sheets by the finite element method. Wei et al. [36] optimized the manufacturing process of carbon fiber reinforced polymer and ethylene–propylene–diene monomer composites by varying the temperature, heating time and type of vulcanizing agent. Lastly, Sobolev et al. [37] reported a new room-temperature synthesis of nanocrystals of scandium-doped zirconium oxide by the sol–gel method.

In this work, we further examine the influence of particle size in the Ag NP ink on the physical characteristics of the sintered structure. A comparative study on the sintering results of various commercial Ag NPs with different particle sizes was performed. After sintering each ink under various temperatures, we observed the differences in the specific resistance, surface morphology, and the resultant particle size. The aim of this study is to discover the influence of the particle size of the Ag NP on the electric characteristics, the final surface morphology, and the particle/grain size of sintered Ag NP inks. Specific resistance was measured using a 4-point probe and a surface profiler, and the surface morphology was observed with field effect scanning electron microscopy (FESEM) and atomic force microscope (AFM) images. The results of this study can be a reference for future manufacturers in selecting the size of the Ag NP and the sintering temperature for processing.

## 2. Experimental Methods

The comparison of the sintering behavior of four kinds of Ag NP inks (ANP Inc., Sejong, Republic of Korea) was carried out. Among them, two kinds of inks had an average particle size of 50 nm, and the other two kinds of inks had an average particle size of 10 nm. The 50 nm inks were commercial inks named DGP-45HTG (400 °C) and DGP-40LT-15C (150 °C), and the 10 nm inks were commercial inks named DGH-55HTG (400 °C) and DGH-55LT-25C (250 °C). The recommended curing temperatures by the manufacturer are presented in parentheses. The dispersant used in the 50 nm inks was triethylene glycol monoethyl ether (TGME), while the 10 nm inks used tetradecane.

Table 1 shows detailed information about the ink. The substrate used in the experiment was Eagle-XG glass (Corning, Corning, NY, USA) with dimensions of 24 × 24 × 0.5 mm^3^ (width × height × thickness). The substrates were immersed in ultrasonic cleaning for 10 min with acetone, and another 10 min in isopropyl alcohol (IPA). After the ultrasonic cleaning, the substrate was heated in an oven at 100 °C for 10 min for baking. The Ag NP ink was spin-coated on the dried substrate with an average layer thickness of 300 nm. The thickness of the spin-coated sample had some minor variations, as was the case in our previous study.

**Figure 1:**
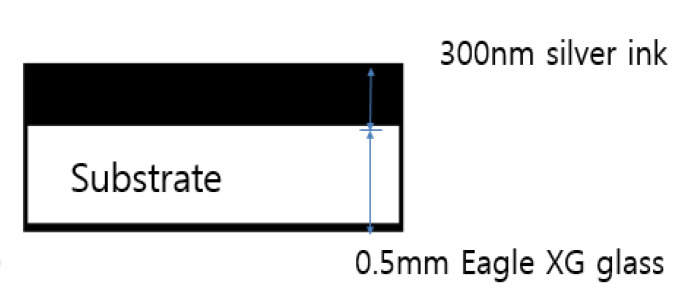
Schematic of a spin-coated conductive ink sample for furnace sintering.

Figure 1 shows the schematics of the Ag NP ink printed on the substrate. The spin-coated sample was first dried on the hot plate at 50 °C for 5 min. After drying, each ink was heated in the furnace (Lab. Companion OF-11E, JEIOTECH, Daejeon, Republic of Korea) for 30 min under the following temperatures: 50, 100, 150, 200, 250, 300, 350, and 400 °C. The sintering time of 30 min was decided based on the results of our previous study, so that the resistivity values could be stabilized with respect to sintering time [21]. The specific resistance of the sintered samples was calculated with the resistance measured through a 4-point probe. Equation (1) is used to calculate the sheet resistance from the resistance obtained through a 4-point probe.

(1) $$R_{S}=4.532\frac{V}{I}.$$

The relationship between the calculated sheet resistance and the specific resistance is as follows [13]:

(2) $$\rho =t\cdot R_{S}$$

where *t* is the thickness of the coated ink. The thickness of the prepared samples was measured three times using a surface profiler. The resistance of each case was measured five times for liability. The FESEM and AFM were used to measure the surface conditions of the sintered samples, as well as to examine the surface conditions of the Ag NP inks sintered under different temperatures. Finally, a thermogravimetric analysis (TGA) was carried out to discover the difference between the sintering behavior of each ink.

## 3. Results and Discussion

**Figure 2:**
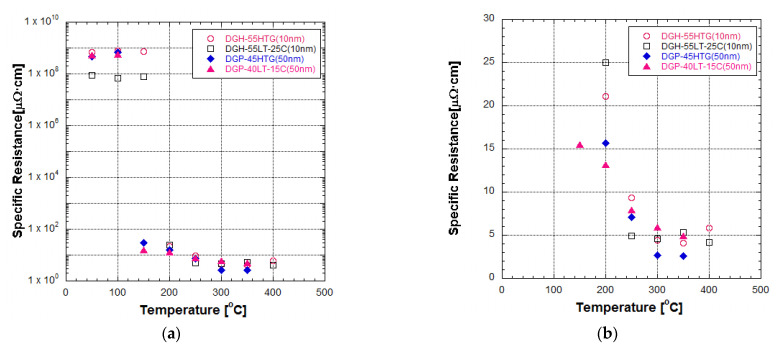
Specific resistance of two different particle size inks after furnace sintering at various temperatures: (**a**) logarithmic scale; (**b**) linear scale.

Figure 2 presents the specific resistance of two different particle size inks after furnace sintering at various temperatures. At low sintering temperatures, the reduction in specific resistance by the increase in sintering temperature was obvious but the specific resistance values remained around the order of 1 to 10 Ω∙m. The specific resistance of DGH-55LT-25C remained a little below 1 Ω∙m. Other Ag NP inks remained a little below 10 Ω∙m. In contrast, the specific resistance of the inks experienced a drastic decrease when the sintering temperature exceeded a certain temperature. In the case of 50 nm inks, this specific resistance drop happened when the sintering temperature increased from 100 °C to 150 °C. The specific resistance of DGP-45HTG dropped from 6.7817 Ω∙m to 30.4 μΩ∙cm, and the specific resistance of DGP-40LT-15C dropped from 5.5911 Ω∙m to 15.4 μΩ∙cm. In the case of 10 nm inks, this specific resistance drop happened when the sintering temperature increased from 150 to 200 °C. The specific resistance of DGH-55HTG dropped from 7.33 Ω∙m to 21.0 μΩ∙cm, and the specific resistance of DGH-55LT-25C dropped from 0.78 Ω∙m to 25.0 μΩ∙cm. Figure 2b shows the linear scale graph of the specific resistance of the inks sintered at temperatures from 200 to 400 °C. We can see from Figure 2b that the decrease rate of specific resistance declined as the sintering temperature increased. After the drastic decrease in specific resistance, the specific resistance of all types of Ag NP inks maintained a decreasing trend until 250 °C. After that, the specific resistance of DGH-55LT-25C remained stable from 250 to 400 °C. The specific resistance of DGH-55HTG decreased until 350 °C but increased at the sintering temperature of 400 °C. The specific resistance of 50 nm inks maintained a decreasing trend until 350 °C, while the specific resistance values corresponding to the sintering temperature of 400 °C was not observable. The lowest specific resistance obtained from this experiment was 2.6 μΩ∙cm in the case of 50 nm ink sintered under 350 °C; this value is about 1.6 times the bulk silver specific resistance of 1.6 μΩ∙cm.

**Figure 3:**
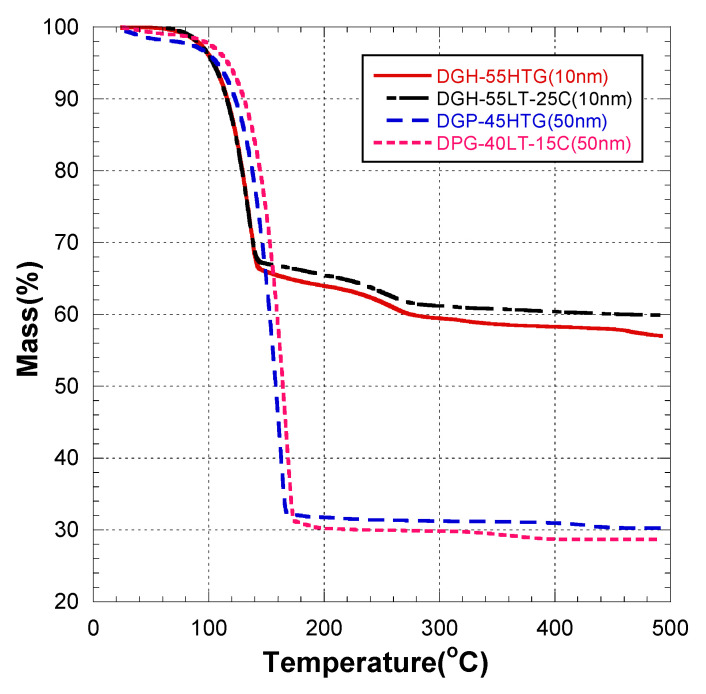
Thermogravimetric analysis of silver nanoparticle conductive inks.

The TGA results of Figure 3 show the reason behind this trend. There was no difference in sintering behavior between the Ag NP inks with the same NP size and different recommended curing temperatures. Generally, the influence of recommended curing temperatures suggested by the manufacturer was insignificant with respect to the specific resistance, compared to the influence of NP size. The mass fraction decrease rate of both 10 nm inks showed abrupt changes at the sintering temperatures of 150 and 250 °C. After 250 °C, the mass fractions of the 10 nm inks were stable. The mass fractions of both 50 nm inks decreased until 180 °C and were relatively stable after 180 °C. Thus, we do not emphasize the recommended curing temperature difference of NP inks of the same size.

**Figure 4:**
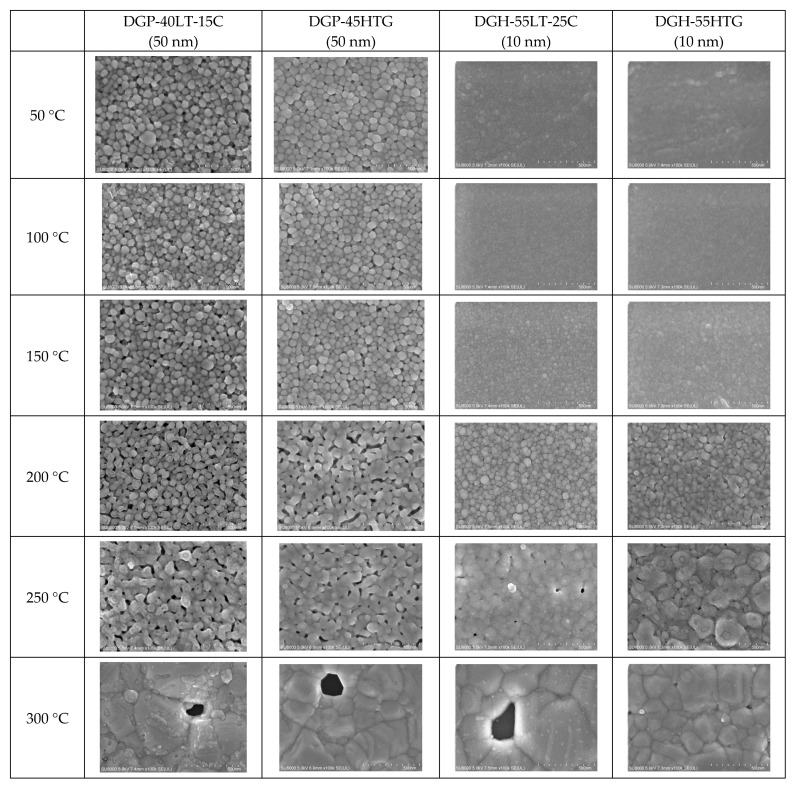

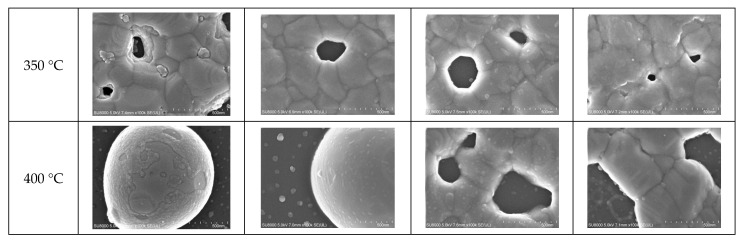
Morphologies of the inks furnace-sintered at various temperatures.

**Figure 5:**
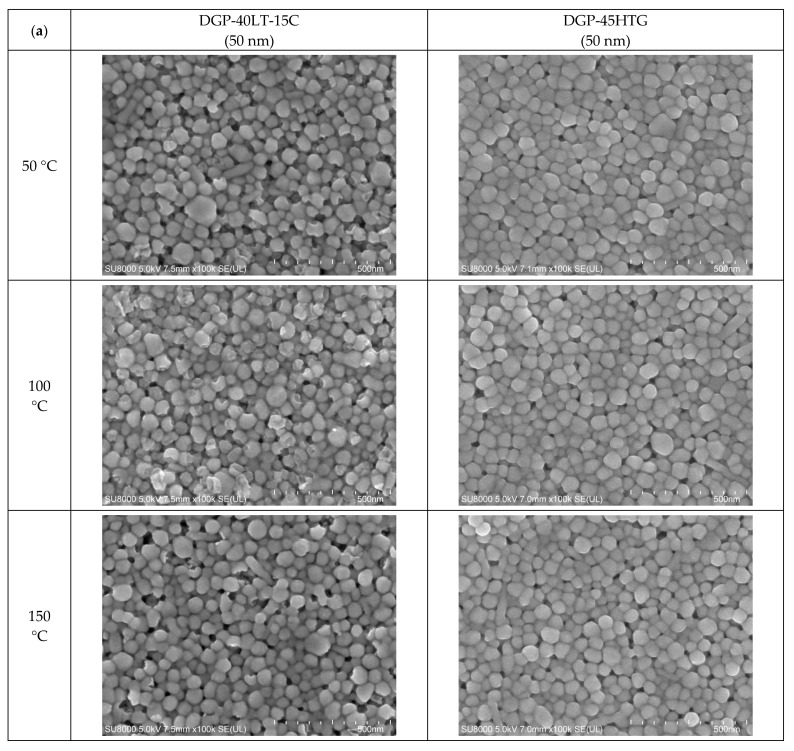

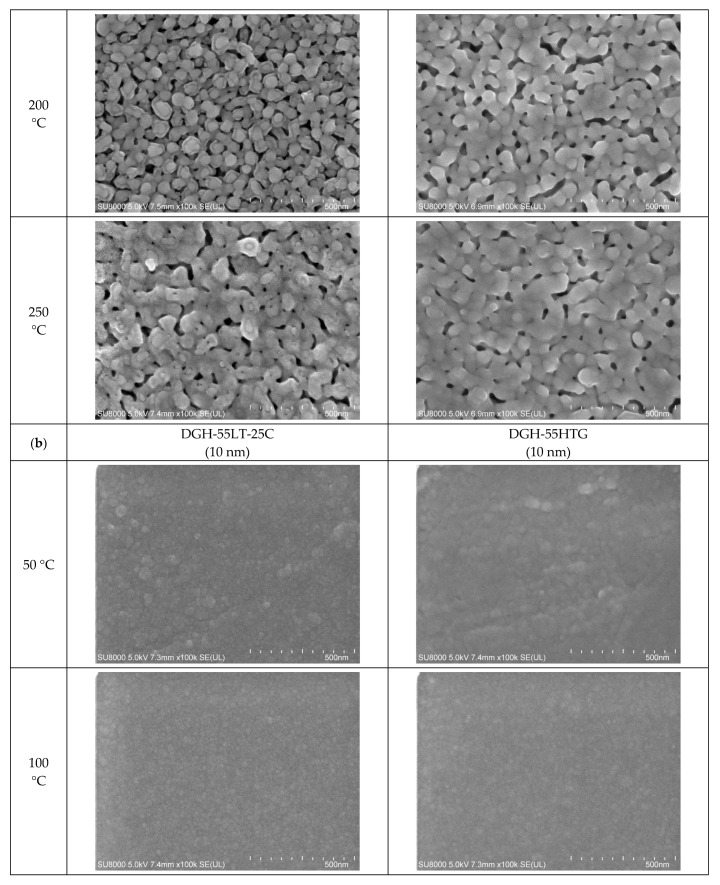

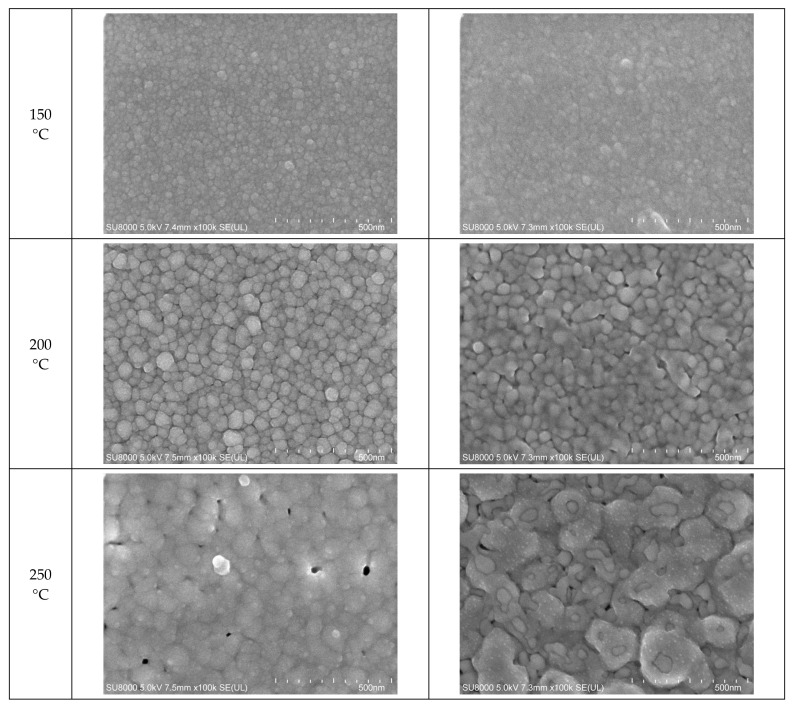
Closer view of the morphologies of the inks furnace-sintered at 50 °C, 100 °C, 150 °C, 200 °C, and 250 °C: (**a**) 50 nm-sized Ag NP; (**b**) 10 nm-sized Ag NP.

**Figure 6:**
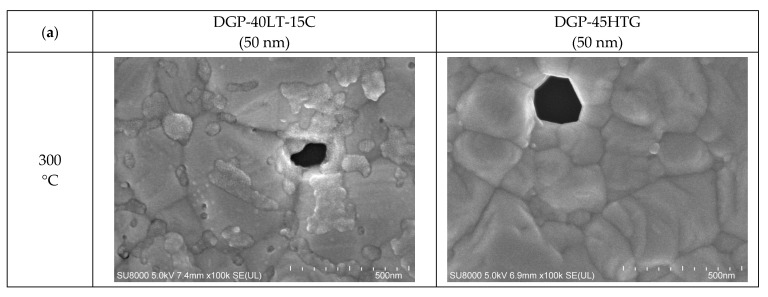

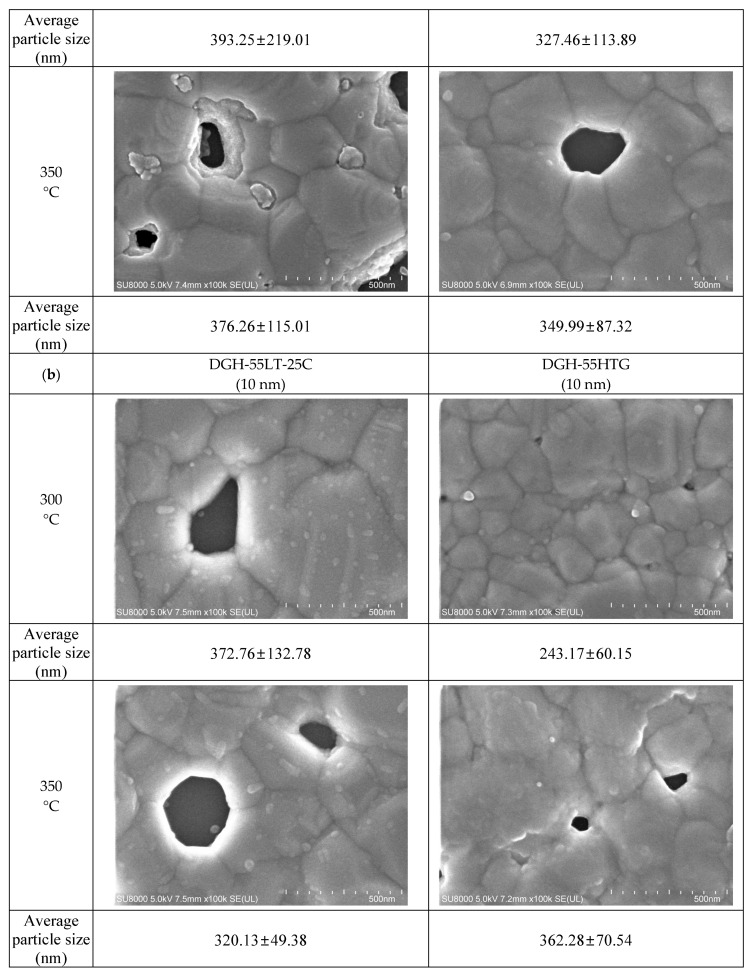

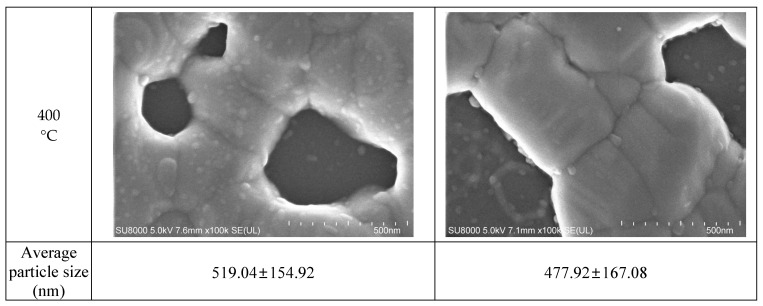
Average particle size measured with FESEM images: (**a**) 50 nm-sized Ag NP furnace-sintered at 300 and 350 °C; (**b**) 10 nm-sized Ag NP furnace-sintered at 300, 350, and 400 °C.

The FESEM images of the sintered Ag NP are shown in Figure 4, Figure 5 and Figure 6. Figure 4 shows the overall view of FESEM images in all the cases. Generally, grain structure was observed from temperatures of 300 °C and above. We have provided a closer view of FESEM images for sintering temperatures from 50 to 250 °C in Figure 5, and a closer view of FESEM images for sintering temperatures of 300 °C and above together with the grain size measurements in Figure 6. The FESEM images show that the resistivity values in Figure 2 correlate with the sintered morphology of Ag NP. The sintered morphology also shows the sintering mechanisms of Ag NP with different particle sizes. From the FESEM images, we can confirm that the temperature rise causes Ag diffusion, initiating neck formation and grain growth at the particle boundaries. These processes induce decreases in specific resistance because they enhance the movement of electrons [38,39]. In the case of 50 nm Ag NP, the particle size did not vary much until the sintering temperature of 200 °C. However, the sintering mechanism of neck growth was observable at the sintering temperatures of 150 and 200 °C. Neck formation is a common sintering mechanism whereby a neck is formed between the Ag NP due to the surface energy reduction caused by Ag diffusion. The neck formation boosts the connection between particles, and leads to densification [6]. In Figure 5a, the neck formation initiation at the sintering temperature of 150 °C was emphasized by marking the corresponding NP. If we associate it with the specific resistance drop of the 50 nm Ag NP ink at this temperature, we can ascertain that these necks free the movement of electrons. At the sintering temperature of 200 °C, neck formation is enhanced and can be easily found. With a higher sintering temperature of 250 °C, we notice neck growth and the agglomeration of Ag NP. Finally, at the sintering temperature of 300 °C, we can see the formation of grain structure in the sintered Ag morphology. We can witness the sintering mechanism of grain growth at the sintering temperature of 350 °C. Grain growth, on the other hand, is a sintering mechanism where the size of Ag grain is increased after the grain formation process [40]. The constant decrease in specific resistance of 50 nm NP from the sintering temperatures from 150 to 350 °C can be associated with the observation of sintering mechanisms at each sintering temperature. Disconnected islands were observed at the sintering temperature of 400 °C, explaining why specific resistance was not measurable at that sintering temperature.

In the images of 10 nm Ag NP ink, the particles do not appear in the figures when the sintering temperature is 100 °C or lower, contrary to the images of higher temperatures. The particles are not observable at the lower sintering temperatures because the particles are too small to detect at these temperatures. The particles appear in the images only when the sintering temperature is 150 °C or higher, where they grow large enough to be noticeable. The size of the 10 nm NP becomes comparable to that of the 50 nm NP from the sintering temperature of 200 °C, where we observe the sudden specific resistance drop of the 10 nm NP ink. The sintering mechanism observed in the lower sintering temperatures from 50 to 250 °C is diffusion on the Ag NP boundary due to a surface energy decrease [41] and Ostwald ripening (the phenomenon where small particles merge to grow into larger particles) due to the surface energy of the heated nanoparticle [42]. Figure 5a,b show a distinct surface phenomenon in the Ag nanoparticle inks with different particle sizes when the sintering temperature is lower than 300 °C. In the case of 50 nm inks, the grain growth usually occurs at sintering temperatures above 300 °C, and the neck formation dominates at sintering temperatures below 250 °C. In contrast, in the case of 10 nm inks, grain growth starts at lower sintering temperatures due to the higher surface energy. For sintering temperatures higher than 250 °C, grain formation and grain growth was observed as in the cases of 50 nm ink. However, at the sintering temperature of 400 °C, isolated Ag NP were not observed as opposed to 50 nm ink. Instead, the presence of pores was noticeable, which explains the increase in specific resistance.

**Figure 7:**
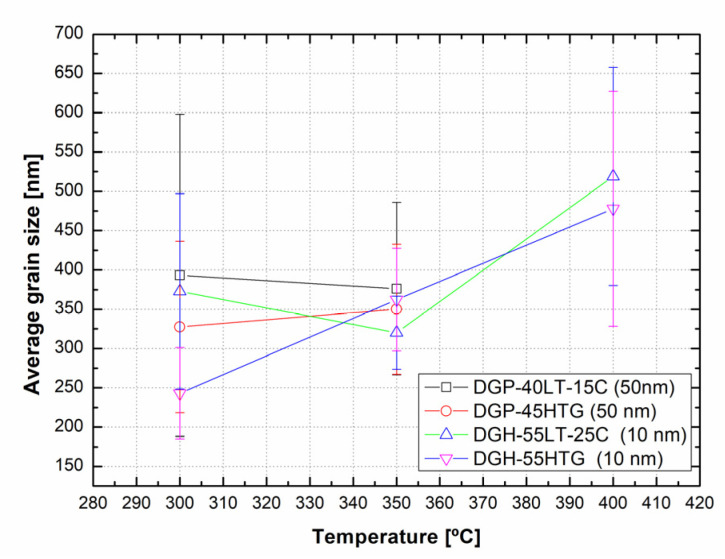
Average grain size vs. temperature (300~400 °C).

Figure 6 presents the grain sizes of the 50 and 10 nm-sized Ag NP sintered at 300 °C and above, based on the FESEM images. Figure 6a shows the 50 nm Ag NP with sintering temperatures of 300 and 350 °C. The size of each grain was measured and labeled using an image processing tool. The averages of grain size of DGP-40LT-15C sintered at 300 and 350 °C were 393.25 and 376.26 nm, respectively. The averages of grain size of DGP-45HTG sintered at 300 and 350 °C were 327.46 and 349.99 nm, respectively. The grain size of the two inks varied with respect to the sintering temperature. It was also noticeable that the standard deviations (SDs) of the grain size decreased for both inks. The SDs of grain size of DGP-40LT-15C sintered at 300 and 350 °C were 219.01 and 115.01 nm, respectively. The SDs of grain size of DGP-45HTG sintered at 300 and 350 °C were 113.89 and 87.32 nm, respectively. This indicates that the smaller grains have grown, and the particles became similar in size. The grain size measurements in Figure 6a indicate that the decrease in specific resistance of 50 nm Ag NP is related to the increase in grain size of the Ag NP. However, the changes in the specific resistance and the grain size were both relatively insignificant compared to the changes in the sintering temperature range of 50 to 300 °C. Figure 6b shows the grain size of the 10 nm NP with sintering temperatures 300, 350, and 400 °C. Figure 7 is the graph of the average grain size with respect to the temperatures from 300 to 400 °C. The averages of grain size of DGH-55LT-25C sintered at 300, 350, and 400 °C were 372.76, 320.13, and 519.04 nm, respectively. The SDs of grain size of DGH-55LT-25C sintered at 300, 350, and 400 °C were 132.78, 49.38, and 154.92 nm, respectively. The averages of grain size of DGH-55HTG sintered at 300, 350, and 400 °C were 243.17, 362.28, and 477.92 nm, respectively. The SDs of grain size of DGH-55HTG sintered at 300, 350, and 400 °C were 60.15, 70.54, and 167.08 nm, respectively. The correlation between specific resistance and grain size for 50 nm Ag NP was not noticeable for the 10 nm Ag NP. Instead, the pore size was the dominant factor for 10 nm Ag NP. At the sintering temperature of 400 °C, we can observe the drastic increase in the pore size in Figure 3 and Figure 6b. The abrupt increase in pore size does not result in a disconnection between grains but does result in a rise in specific resistance. The drastic increase in pore size restricts the paths for electron movement.

**Figure 8:**
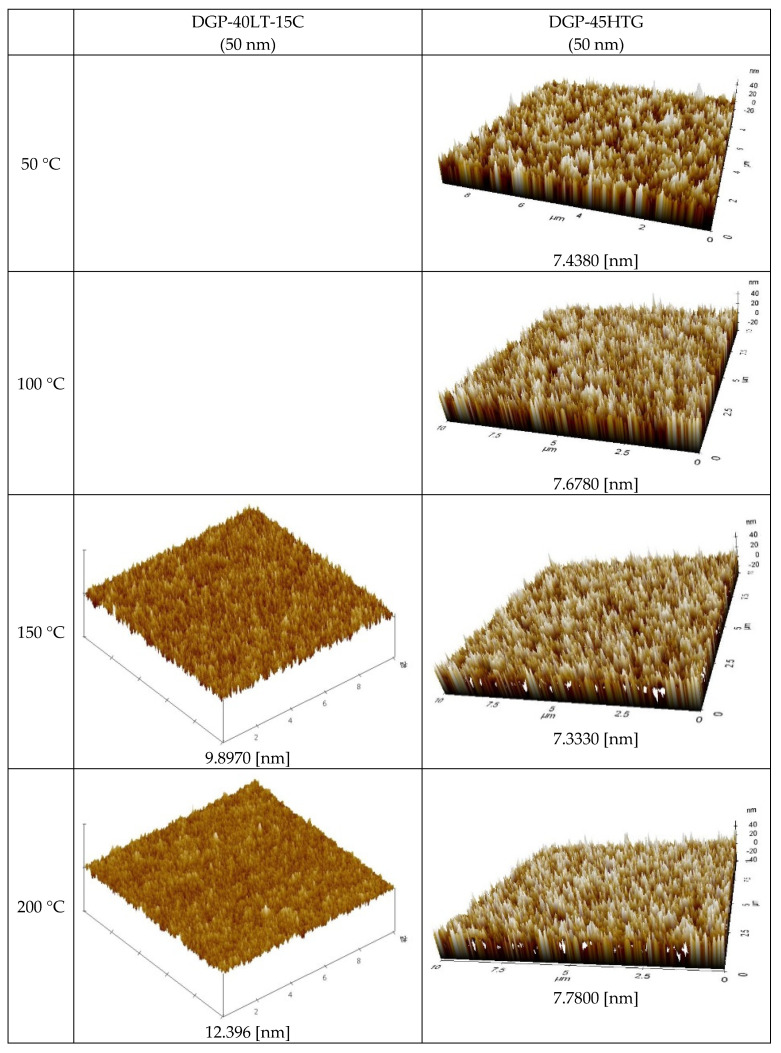

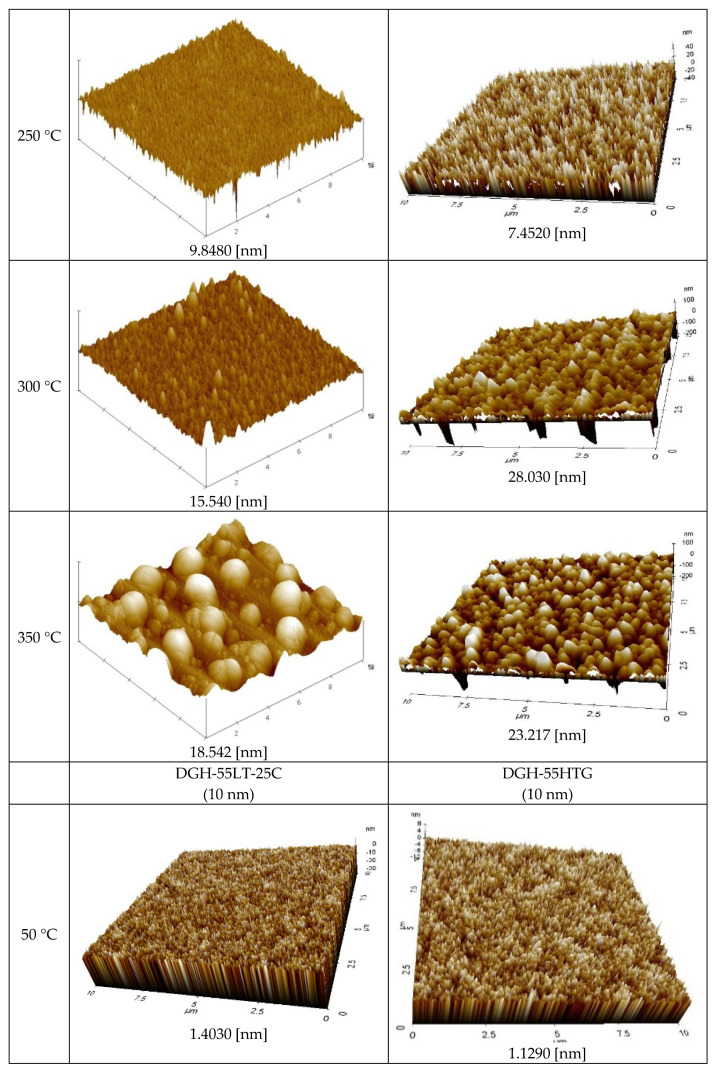

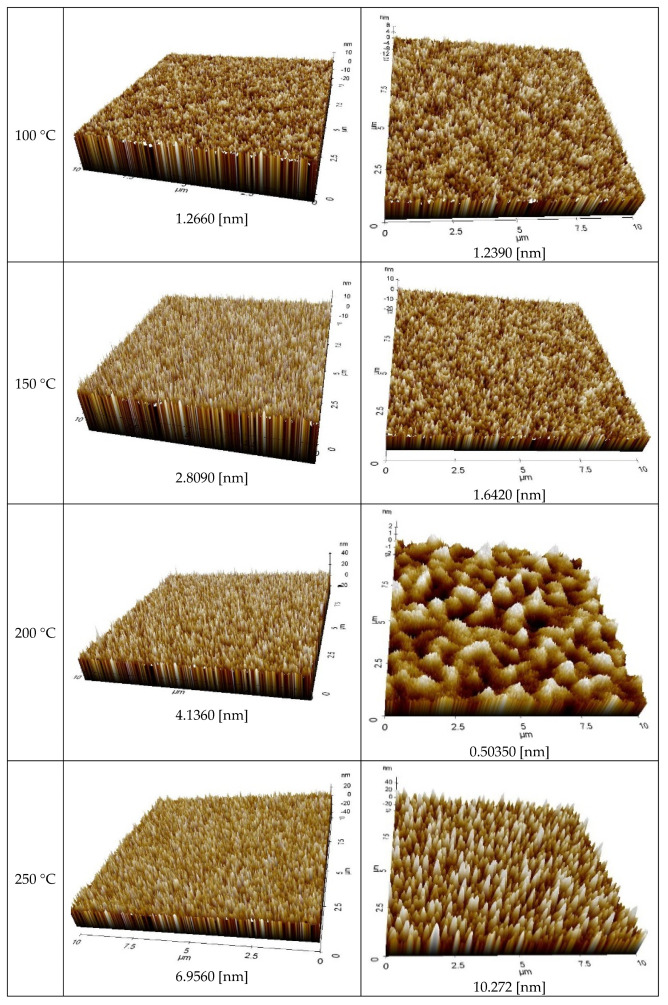

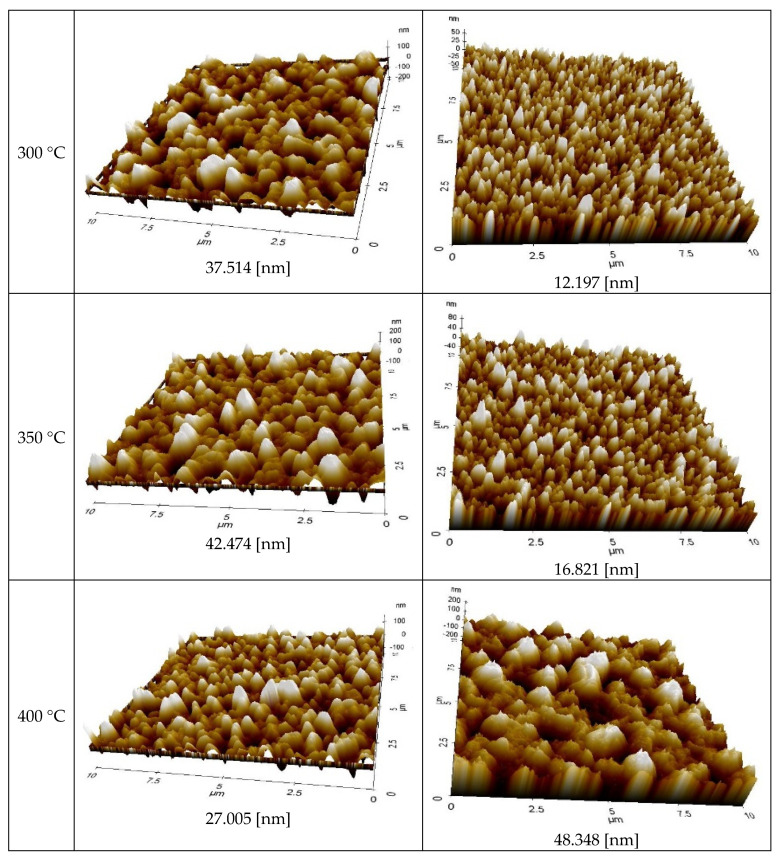
AFM images of the inks furnace-sintered at various temperatures.

**Figure 9:**
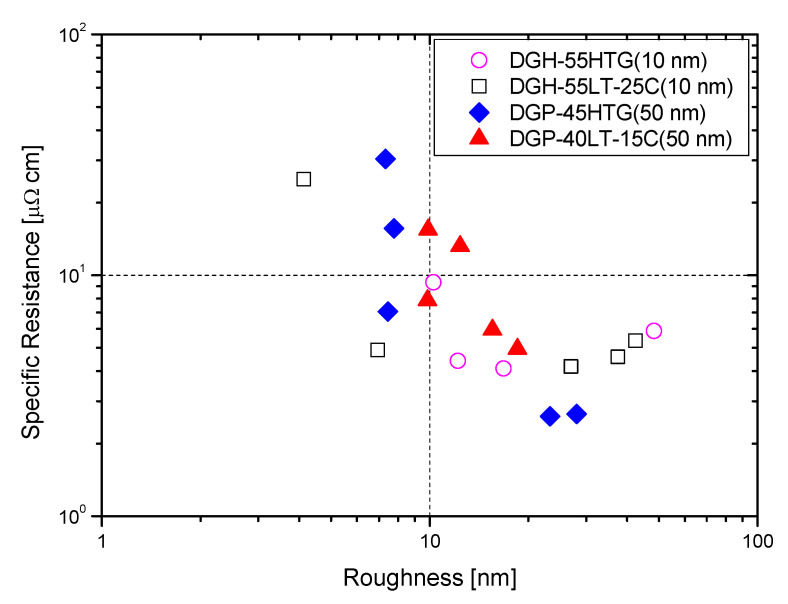
Specific resistance vs. roughness.

Figure 8 displays the AFM measurement results that show the roughness of the Ag NP ink with respect to sintering temperature. Generally, the roughness of the Ag NP increased as the sintering temperature increased. The FESEM images in the cases with various temperatures show that both pore size and grain size of the Ag NP ink increase as the sintering temperature increases. The excessive growth of grains could lead to increases in the roughness, and this could affect the rise in specific resistance. At lower sintering temperatures, the roughness values of Ag NP are generally below 10 nm. At lower sintering temperatures, 50 nm Ag NP had larger roughness, compared to 10 nm Ag NP, which was to be expected, due to the Ag NP size differences. The roughness of Ag NP starts to be larger than 10 nm with a sintering temperature of 300 °C or higher. The roughness values of 50 and 10 nm-sized Ag NP become similar after the sintering temperature exceeds 250 °C, but the roughness values higher than 30 nm are only observed in 10 nm Ag NP. Figure 8 shows the correlation between specific resistance and roughness for cases with electrical conductance. It can be seen from Figure 9 that when the roughness value is below 20 nm, a higher roughness generally indicates a lower specific resistance. This can be associated with grain growth. However, when the roughness value exceeds 30 nm, the relationship between specific resistance and roughness is reversed. For example, the roughness of DGH-55LT-25C follows the trend of specific resistance. The roughness was 37.514, 42.474, and 27.005 nm at 300, 350, and 400 °C; the specific resistance was 4.58, 5.34, and 4.17 μΩ∙cm at 300, 350, and 400 °C. We can see that the increase in porosity due to excessive growth of grain was reflected by both the specific resistance and the surface roughness. This shows the correlation between specific resistance and surface roughness. In other words, the increases in grain size and pore size due to grain growth influences the increase and decrease in the Ag nanoparticle ink specific resistance, and the changes in the surface roughness confirm this phenomenon.

## 4. Conclusions

This study has confirmed the physical characteristics and the surface phenomenon of Ag NP inks with different particle sizes during the sintering process at various temperatures. The findings of this study can aid future manufacturers in deciding the particle size and sintering temperature for their applications. After consideration of these experiments, our conclusions are summarized below.

The specific resistance of the 50 nm Ag NP ink dropped significantly at temperatures higher than 150 °C; the specific resistance of 10 nm Ag NP ink dropped significantly at temperatures higher than 200 °C. The specific resistance tended to decrease with the increase in sintering temperature, regardless of whether this occurred before or after the significant drop in specific resistance. However, over sintering of Ag NPs at high sintering temperatures could lead to an increase in specific resistance due to pore growth and crack growth by thermal expansion.

From the comparison of FESEM images and specific resistance, we were able to determine the differences in the sintering mechanism with different particle sizes of Ag NP ink. The mechanism of specific resistance decrease in 50 nm Ag NP inks was neck formation in the boundary of the Ag NP. In contrast, the mechanism of specific resistance decrease in 10 nm Ag NP inks was the growth of the NPs.

The grain growth of 50 nm Ag NP ink started from the sintering temperature of 300 °C. In contrast, in the case of 10 nm Ag NP ink, smaller NPs led to more rapid sintering, and the grain growth was noticeable from 200 °C.

The surface roughness measurements through AFM reflected increases in grain size and pore size due to grain growth. At surface roughness values below 20 nm, a higher surface roughness tended to be associated with an increase in particle size and a lower specific resistance. On the other hand, at surface roughness values higher than 30 nm, a higher surface roughness tended to be associated with an increase in pore size and a higher specific resistance. The Ag NP samples with the best specific resistance values had a surface roughness value within the 20~30 nm range.

## Figures and Tables

**Table 1:** Information on silver nano particle inks.

	DGP-40LT-15C (50 nm)	DGP-45HTG (50 nm)	DGH-55HTG (10 nm)	DGH-55LT-25C (10 nm)
Ag content (wt%)	31	32	56	58
Particle size (nm)	~50	~50	~10	~10
Viscosity (cPs)	10~17	18.2	9.75	8.0
Surface tension (mN·m^−1^)	36	37.4	29.2	29.2
Solvent	TGME	TGME	Tetradecane	Tetradecane
Curing temp. (°C)	150 °C	400 °C	400 °C	250 °C
Specific resistance (μΩ·cm)	11~12	2~3	2.0~2.5	2.4~3.0
Substrate	Eagle XG glass	Eagle XG glass	Eagle XG glass	Eagle XG glass

## Data Availability

The original contributions presented in the study are included in the article; further inquiries can be directed to the corresponding author.
